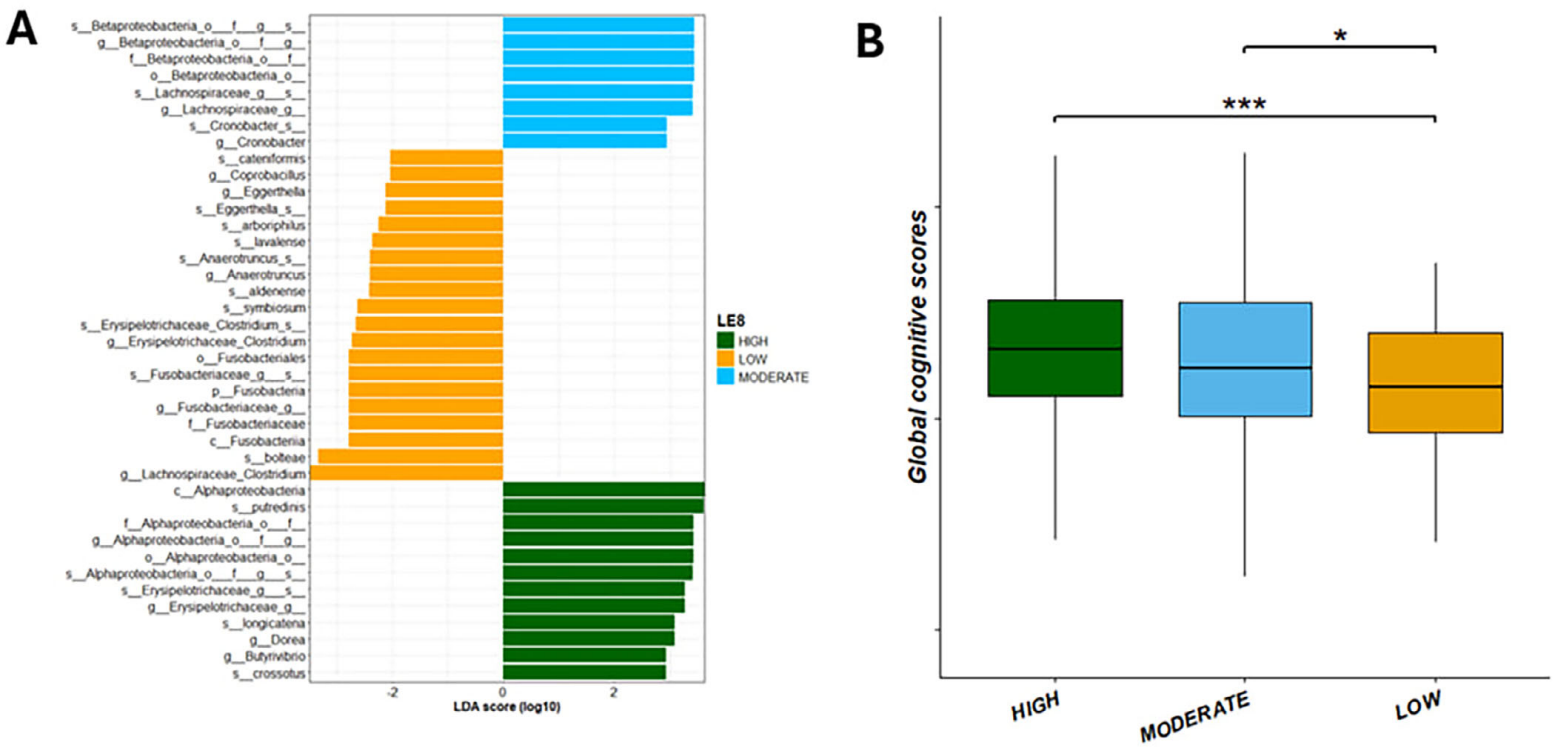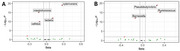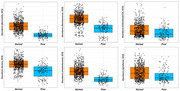# Sleep health influencing gut microbiome showed an association with cognitive performance

**DOI:** 10.1002/alz70860_096594

**Published:** 2025-12-23

**Authors:** Ireneaus Nyame, Yannick Joel Wadop Ngouongo, Biniyam A. Ayele, Alice Nono Djotsa, Xueqiu Jian, Alfred N. Fonteh, Sudha Seshadri, Jayandra Jung Himali, Bernard Fongang, The African Initiative on Bioinformatics Online Training in Neurodegenerative Diseases (AI‐BOND)

**Affiliations:** ^1^ University Of Cape Coast, Cape Coast, Central, Ghana; ^2^ Glenn Biggs Institute for Neurodegenerative Diseases, University of Texas Health Science Center at San Antonio, San Antonio, TX, USA; ^3^ Global Brain Health Institute, San Francisco, CA, USA; ^4^ John P. Hussman Institute for Human Genomics, University of Miami Miller School of Medicine, Miami, FL, USA, Miami, FL, USA; ^5^ Addis Ababa University, Addis Ababa, Addis Ababa, Ethiopia; ^6^ Section of Health Services Research, Baylor College of Medicine, Houston, TX, USA; ^7^ The University of Texas Health Science Center at San Antonio, San Antonio, TX, USA; ^8^ University of Texas Health Science Center at San Antonio, San Antonio, TX, USA; ^9^ Huntington Medical Research Institutes, Pasadena, CA, USA; ^10^ Glenn Biggs Institute for Alzheimer's & Neurodegenerative Diseases, San Antonio, TX, USA; ^11^ The Framingham Heart Study, Framingham, MA, USA; ^12^ Department of Population Health Sciences, University of Texas Health Sciences Center, San Antonio, TX, USA; ^13^ Glenn Biggs Institute for Alzheimer's & Neurodegenerative Diseases, University of Texas Health San Antonio, San Antonio, TX, USA; ^14^ Boston University School of Public Health, Boston, MA, USA; ^15^ Glenn Biggs Institute for Alzheimer's & Neurodegenerative Diseases, The University of Texas Health Science Center at San Antonio, San Antonio, TX, USA; ^16^ Department of Population Health Sciences, The University of Texas Health Science Center at San Antonio, San Antonio, TX, USA; ^17^ Department of Biochemistry and Structural Biology, The University of Texas Health Science Center at San Antonio, San Antonio, TX, USA

## Abstract

**Background:**

The American Heart Association has identified eight metrics for improving heart and brain health, including sleep health. Recent studies highlight the strong link between sleep health, gut microbiome, and diseases like Alzheimer's. While sleep deprivation is known to affect gut microbiome and brain health, the specific impact of sleep health on gut microbiome and cognitive disorders remains largely unexplored.

**Method:**

We analyzed stool samples and sleep metrics from 781 participants (mean age 54.9, 57.1% female) in the Framingham Heart Study to examine the effect of sleep on gut microbiome composition and cognitive performance. Using the V4 region of the 16S rRNA and Lefse analysis, we identified microbiome profiles related to sleep health. ANOVA assessed the sleep‐cognitive performance relationship, while multivariable and differential abundance analyses explored the microbiome's link to cognitive function, controlling for age, sex, and education.

**Result:**

Differences in bacterial diversity were observed between low, moderate, and high groups. Lefse analysis showed higher levels of aldenense, bolteae, symbiosium, and lavalense in the low group, while Butyrivibrio, putredinis, and Dorea were less abundant. ANOVA indicated a significant correlation between global cognitive scores and sleep metrics (*p* = 0.0024). Positive correlations were found between cognitive scores and Pseudobutyrivibrio and Ruminococcus, while negative correlations were observed with Barnesiella and Clostridium. At the species level, xylanivorans and lactaris were positively correlated, whereas boltea, callidus, and intestinihominis were negatively correlated with cognitive scores.

**Conclusion:**

Our findings showed that individuals with good sleep scores had higher cognitive performance, while those with lower sleep scores had lower cognitive performance. The results also indicated an association between gut microbiome and sleep metric as well as between gut microbiome and cognitive performance. Finally, our work revealed that the taxa *Clostridium* and *bolteae* exhibited association with both sleep metric and cognitive performance. Further studies should be conducted to understand the effects of sleep metric on the relationship between gut microbiome and the risk of developing Alzheimer's Disease and Related Dementias (ADRD).